# Analysis of the curative effect of percutaneous kyphoplasty in the treatment of osteoporotic vertebral compression fracture with intravertebral clefts

**DOI:** 10.1097/MD.0000000000025996

**Published:** 2021-06-04

**Authors:** Dongfang Li, Yingjie Zhou, Hongxun Cui, Liang Kong, Wenxiao Zhu, Xubin Chai, Hanjie Zhuo

**Affiliations:** Department of Spine Surgery, Luoyang Orthopedic Hospital of Henan Province, South Qiming Road, Luoyang, China.

**Keywords:** osteoporosis, percutaneous kyphoplasty, spinal fractures

## Abstract

Kummell's disease is a delayed vertebral collapse fracture caused by posttraumatic osteonecrosis. It is a special type of osteoporotic vertebral fracture in the elderly. This study compares and analyzes the difference in the curative effect of 2 kinds of osteoporotic vertebral compression fracture (OVCF) in the presence of fracture or not in the vertebral body, and provides a clinical reference for the application of percutaneous kyphoplasty (PKP).

This research is a kind of retrospective analysis from January 2012 to January 2015, PKP was used to treat 165 patients with osteoporotic vertebral compression fracture. The patients were divided into 2 groups: Intravertebral clefts group (group A) and none-intravertebral clefts group in vertebral body (group B). Bone mineral density (BMD), bone cement injection (BCI), Visual analogue scale (VAS) score before and after surgery, anterior, central and posterior height of vertebral body (before and after surgery) and Cobb angle of injured vertebra (before and after surgery) were compared between the 2 groups.

Surgeries for 165 patients in the 2 groups were successfully completed, and 226 fractured vertebrae were performed through bilateral puncture approach to strengthen the vertebral body. Intraoperative injection of bone cement (ml) was 4.25 + 1.29 (range: 2.6–7.8). There were statistically significant differences in bone cement injection quantity between the 2 groups (*P* < .05), and in bone cement leakage (*P* > .05) as well as the Postoperative VAS score (*P* < .05). However, There was no statistical difference in VAS score before surgery between the 2 groups (*P* > .05). The results indicated that the pain relief degree of OVCF patients without intravertebral clefts is better than that in the vertebral body. No statistical difference was found in Cobb Angle before and after surgery (*P* > .05), as well as the correction rate of the injured vertebrae before and after surgery (*P* > .05). There was no statistical difference in the degree of recovery of the anterior, middle and posterior margins of the injured vertebrae after surgery (*P* > .05).

PKP treatment led to better degree of pain relief in OVCF patients without intravertebral clefts, and less bone cement was injected into the surgery.

## 1 Introduction

### 1.1 Background

Almost all fractures in the clinic can be healed after 6 to 7 weeks, and there is no need for surgery In 1895, German surgeons first described a special type of osteoporotic vertebral compression fracture (OVCF).^[1]^ For many years, The OVCF with intravertebral clefts attracted the attention of many scholars, which also known as vertebral ischemic necrosis, Kummell's disease, etc. The OVCF with intravertebral clefts is a chronic and non-healing OVCF, which is related to the occurrence of ischemic necrosis in the vertebral body after fracture, and occurs in the continuous collapse of vertebral body after minor trauma.^[2,3]^ Kummell's disease is different from general OVCF and should be differentiated. General OVCF more than after injury or there are certain inducements, after rest braking its pain can be gradually alleviated. In subacute stage, bone marrow edema and X-ray and CT examination showed that there were no other abnormal changes except vertebral fracture. Kummells disease could be attributed to MRI. Its pathogenesis and pathological mechanism were different from those of general OVCF, the course of disease was long, and it was difficult to recover from low back pain. Progressive deterioration of vertebral collapse. The percutaneous kyphoplasty (PKP) treatment vertebral body fracture OVCF with lower leakage of bone cement and protrusion deformity after corrective action.^[4–6]^ In this study, a comparative study was conducted to compare the treatment of 2 kinds of OVCF with or without intravertebral clefts, providing a clinical reference for the application of PKP.^[7,8]^

### 1.2 Hypothesis

PKP is used to treat the presence of OVCF in the vertebral body has the effect of reducing bone cement leakage.

## 2 Methods

### 2.1 The general information of the patients

**Figure 1:**
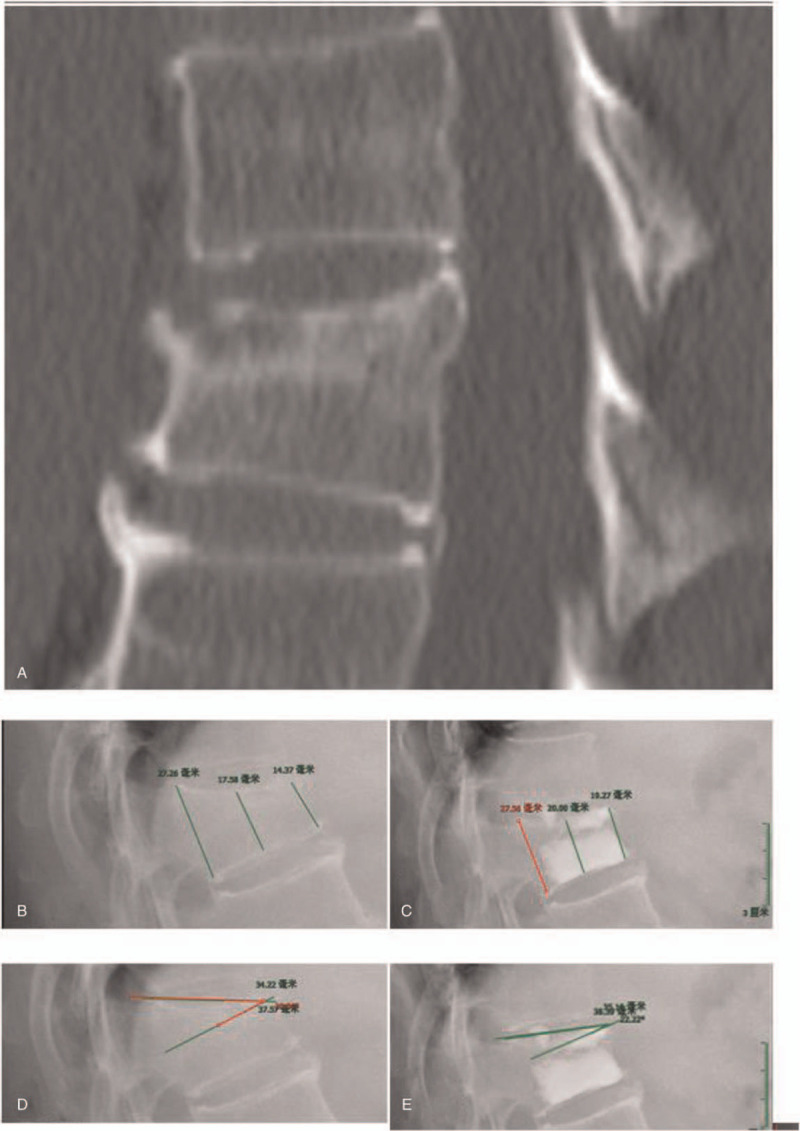
Female, 56 years old (A) non-fissure T12 vertebral body (B) Preoperative sagittal spine showed anterior/ middle/ posterior vertebral height 14.37 mm/ 17.58 mm/ 27.26 mm (C) postoperative anterior/ middle/ posterior vertebral body height 19.27 mm/ 20.00 mm/ 27.56 mm (D) preoperative cobb angle 30° (E) postoperative cobb angle 22.22°.

**Figure 2:**
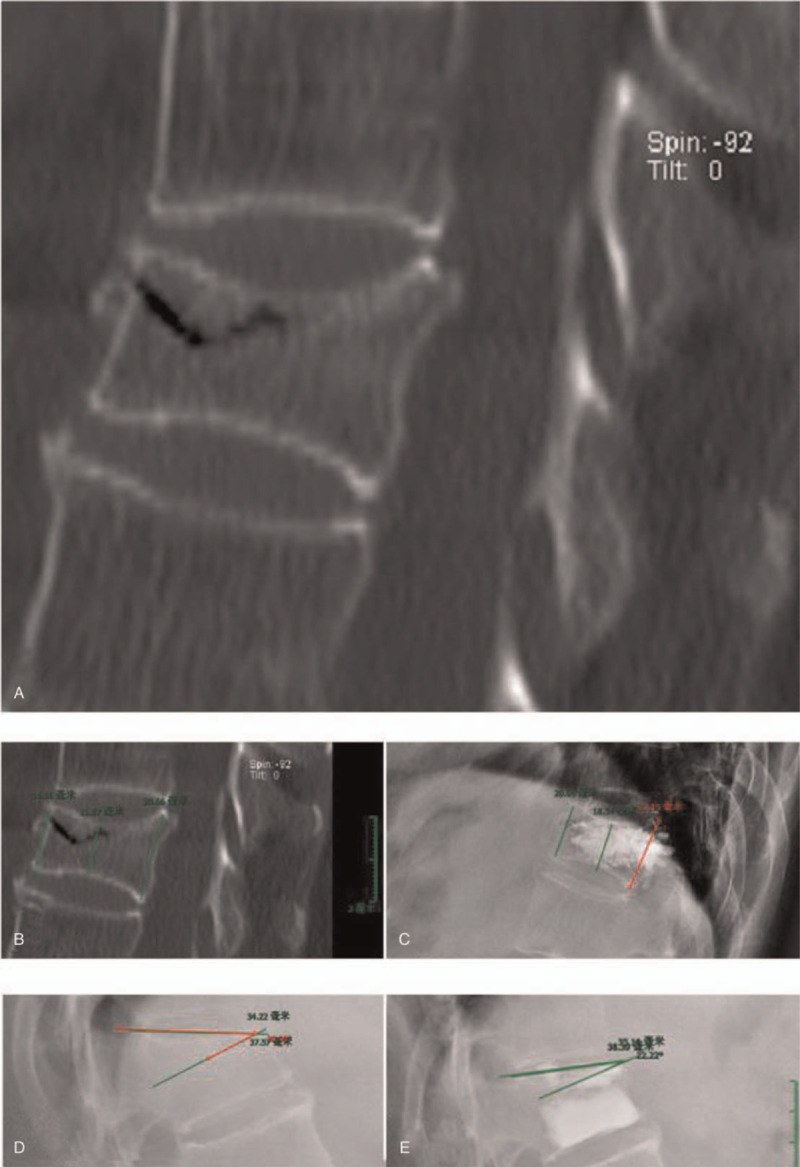
Female, 71 years old (A) fissure T11 vertebral body (B) preoperative anterior/ middle/ posterior vertebral height 14.56 mm/ 11.87 mm/ 20.66 mm (C) postoperative anterior/ middle/ posterior vertebral body height 20.04 mm/ 18.54 mm/ 27.15 mm (D) preoperative cobb angle 16.38° e postoperative cobb angle 9.41°.

A retrospective study was conducted from January 2012 to January 2015. There are 165 patients with 226 fracture vertebral bodies, which were divided into 2 groups: OVCF with intravertebral clefts (group A, 67 cases) and the OVCF without intravertebral clefts (group B, 98 cases). The age of all cases was 69 ± 2.3 (range: 57–92), including 126 female patients (76.3%). All patients in this group had different degrees of osteoporosis. The bone density (T value) is −3.823 ± 1.29 (range:−1–5.8). The vertebral body of the fracture is mostly located in the thoracolumbar segment. There were 139 lesions in the T11-L2 segment, which accounted for 61.5%. Preoperative imaging examination included orthotopic X-ray, CT reconstruction and MRI examination of the fracture site. The MRI examination included T1 weighted image, T2 weighted image, and fat suppression sequence. The imaging changes of the vertebral body without clefts in the vertebral body are shown in Figure 1. CT imaging of intravertebral clefts fracture in vertebral body can be seen in the vertebral body shell sample change. (Fig. 2).

### 2.2 Surgical methods of the PKP surgery

The patient is prone on the surgical bed, local anesthesia. The vertebral body was confirmed by the anterior and posterior position of the c-arm X-ray machine, and the skin puncture point was determined according to the surface projection of the vertebral pedicle. Puncture sagittal plane and coronal plane angle were determined according to fracture line position adjustment of vertebral fracture. Gradually, the puncture needle was pushed forward, and the position of the puncture needle was confirmed. (there was a fracture of the vertebral body and the puncture needle was placed in the crack). Lateral perspective puncture needle tip to the posterior margin of the vertebral body 3 to 5 mm in front of the vertebral arch. Then, under the perspective of monitoring, the balloon is placed into the balloon and the balloon is located in the first 1/3 of the vertebral body. Expand the balloon under fluoroscopy, balloon dilation need to be stopped in such situation: the fracture vertebral body is highly restored; the vertebral body height has not recovered but the balloon has reached the vertebral endplate. expand the balloon in the maximum capacity or the maximum pressure. The bone cement is used to inject bone cement into the vertebral body when the bone cement is in the late stage. We need to observe the dispersion and fill of the bone cement, confirm that the cement is diffused enough to stop the injection. After waiting enough time to confirm the solidification of bone cement, we should turn, pull out the working sleeve and suture skin puncture point.

### 2.3 Evaluation indexes of the PKP surgery

The loss height of the vertebral injury = the average height of the injured vertebra adjacent to normal vertebral body-The preoperative height of injury. The recovery height of the vertebral injury = the height of the postoperative injury-The preoperative height of injury. The degree of the recovery height = The recovery height of the vertebral injury/The loss height of the vertebral injury × 100%.

The Cobb Angle and the correction rate. The postoperative lateral X-ray was used to measure the posterior convex angle of the injured vertebra and assess the severity of kyphosis. The correction rate of posterior kyphosis = (preoperative posterior convex Angle of injury-postoperative convex Angle of injury)/ preoperative posterior convex Angle of injury.

Visual analogue scale (VAS). The pain visual analogue scale was evaluated for the degree of pain before and after surgery.

### 2.4 Statistical analysis

The data were expressed as mean ± standard deviation of separate experiments. The unpaired *t*-test was used to value the difference between groups. The differences in VAS and ODI scores between the 2 groups were used to match the symbol rank and test of the matched design data. Statistical significance was set at p value less than 0.05. Analysis was performed using SPSS version 21.0 (SPSS Inc, Chicago, IL).

## 3 Result

Surgery for 2 groups patients were successfully completed, and the incision healed well. PKP was successfully completed through 226 fractures bilaterally punctured in 165 patients. The bone cement during the surgery was (4.25 ± 1.29) ml (Range: 2.6–7.8). The comparison between the patients with intravertebral clefts (group A) and patients without intravertebral clefts (group B) as following:

1. There were statistically significant differences in bone cement injection between the 2 groups (*P* = .04). However, there was no statistical difference in bone cement leakage. (Table 1).
2. There was no statistical difference in VAS scores between the 2 groups (*P* = .07) before the surgery. While statistically significant differences in VAS scores between the 2 groups after surgery and at the last follow-up (*P* = .04). It indicates that the pain relief degree of the B group is better than A group (Table 1).
3. There was no statistical difference between the 2 groups in the preoperative Cobb Angle (*P* = .06), as well as the Cobb Angle at the end of postoperative and postoperative follow-up (*P* = .07), and 2 groups of patients with kyphosis rate after surgery and at the last follow-up (*P* = .06)(Table 2).
4. There was no statistical difference in the degree of recovery of vertebral body height after surgery between 2 groups (*P* = .06) (Table 3).

**Table 1:** Bone cement injection quantity,Bone mineral density(T value) and VAS score of patients in 2 groups.

	Bone	Bone Mineral	Bone	VAS score		
	Cement	Density	Cement	Before	After	Final
	Injection (ml)	*T* value (SD)	Leakage (case)	surgery	surgery	follow-up
Group A	6.24 ± 2.13	-4.62 ± 0.93	12	8.12 ± 1.23	2.13 ± 1.2	2.13 ± 0.62
Group B	5.15 ± 2.12	-4.71 ± 0.82	15	7.26 ± 1.12	1.53 ± 1.12	1.63 ± 0.31

**Table 2:** Cobb Angle and Kyphosis correction rate of patients in 2 groups.

	Cobb Angle (before surgery)	Cobb Angle (after surgery)	Cobb Angle (Final follow-up)	Kyphosis correction rate (after surgery)	Kyphosis correction rate (Final follow-up)
Group A Group B	12.3 ± 4.3	7.6 ± 4.7	9.3 ± 5.4	0.6 ± 0.2	0.4 ± 0.1
	11.3 ± 5.4	8.6 ± 5.4	10.1 ± 6.3	0.4 ± 0.3	0.3 ± 0.1

**Table 3:** The recovery degree of height of anterior, central and posterior vertebral body in 2 groups.

	Anterior	Central	Posterior
Group A	0.51 ± 0.42	0.32 ± 0.23	0.41 ± 0.12
Group B	0.53 ± 0.41	0.45 ± 0.13	0.54 ± 0.23

## 4 Discussion

The recent researchers believe that the existence of vertebral body fracture OVCF is due to osteoporosis fracture after vertebral fractures caused by ischemic osteonecrosis fracture healing, or even a combination of vertebral body collapse, leading to the pseudarthrosis formation. Laloux's research suggested that there may be 2 reasons for the bone ischemic necrosis of the vertebral body:

1. The bone marrow vessels in the vertebral body have been subjected to compression and stimulation for a long time.
2. The micro fracture on the basis of osteoporosis causes small artery injury in the vertebral body.

The authors suggest that patients with osteoporosis and bone fracture repair ability is poor, and unreasonable brake after vertebral fracture, fracture end abnormal activity is excessive, affect the blood supply of vertebral fracture, bone trabecular ischemic necrosis, leads to the formation of a vertebral body collapse, pseudarthrosis. For the treatment of patients with OVCF in the vertebral body, in order to relieve the pain and correct deformity of the patients, the researchers reported the treatment of bone graft and internal fixation in the vertebral body,^[9]^ and achieved satisfactory results. However, due to osteoporosis, the risk of internal fixation failure and bone graft absorption is higher. The authors suggest that this is not the first choice for such patients. Martin's research^[10]^ shown that PVP treatment of the 2 groups found that PVP had limited effect on the reduction of vertebral injury. Other researchers reported that PKP can restore the height of vertebral body and correct kyphosis and improve the quality of life of patients.^[11–13]^ All cases in our research were treated with PKP. There was no statistical difference between the 2 groups before and after the surgery, as well as the rate of posterior kyphosis after surgery, and the degree of recovery of the anterior margin, middle and posterior margin of the injured vertebrae was observed in the 2 groups. However in our research, there was a statistically significant difference in the degree of recovery of the injury at the end of the last follow-up. It indicates that the vertebral body height of OVCF patients with intravertebral clefts is better. Some scholars^[14]^ reported that patients with PKP were able to better recover the height of the injured vertebra and the correction of kyphotic deformity after treatment with the reduction of the balloon. The authors consider that this may be due to the existence of obvious abnormal activities in the fractured vertebral with clefts, and the height recovery of vertebral injury in the expansion of the balloon.

There was a statistical difference between the 2 groups in bone cement injection. Compared with the OVCF patients without internal clefts, more bone cement was injected into the OVCF patients with internal clefts. So we suggest that this may be related to the clefts formed after the injury of the injured vertebra. The main reason for the pain relief is the disappearance of the abnormal activity of the fracture and the stability in mechanics. The bone cement can stabilize the fracture end and prevent its occurrence of micro - motion, thereby reducing the pain stimulation of the nerve.^[15]^ Some researchers report that the entire vertebra must be filled with bone cement. However, other researchers^[16]^ reported that the amount of bone cement injection ranged from 2.2 to 11.0 ml in patients with treatment, and the results showed that the pain relief was satisfactory in most patients. Barr^[17]^ studied thoracic vertebrae cement injection 2 to 3 ml, and lumbar bone cement injection of 3 to 5 ml, which can cause most of the pain of 97% of patients to be alleviated or even disappeared. Some scholars have found that the strength of vertebral body can be recovered only by 2 ml of bone cement, but the stiffness recovery requires a lot of bone cement (4–8 ml). However, stiffness is the most closely related mechanical parameter with pain relief. We suggest that the microfluidity of the fracture is related to the patient's pain. In combination with the previous study, we believes that the reduction of acute pain in patients is not related to the amount of bone cement, and only the bone cement dispersion can stabilize the fracture terminal at the fracture line and around it. This can also reduce the pain caused by micro - motion of fracture. However, with the extension of follow-up time, the injured vertebra could collapse in different degrees. Kim's research^[18]^ considered that the fracture of the cancellous bone area between the bone cement interface and the upper and lower endplates due to the long-term stress was the main reason for the fracture. Therefore, the author believes that bone cement can be fully disseminated in the vertebra after preventing the leakage of bone cement. The bone cement injected into the spinal cord should be scattered throughout the endplate, reducing the occurrence of the refracture of the responsible vertebral body, and the long-term prognosis of the patients may be better.

The common complication of PKP is bone cement leakage.^[19,20]^ Usually, there is no clinical symptom of bone cement leakage, but individual patients may cause serious complications.^[21]^ By postoperative CT evaluation, this study found no of the spinal cord and nerve root symptoms caused by the leakage of bone cement or pulmonary embolism, mainly appears vertebral side of soft tissue and intervertebral leakage, no statistical differences between the 2 groups. Prevention of bone cement leakage is the key. Before surgery, the imaging data of patients were carefully evaluated, and the fracture of vertebral wall and vertebral body were well understood as well as the selection of puncture point and puncture Angle. Bone cement is injected into the bone cement during the late stage or in the early mass stage, and the maximum possible prevention of bone cement leakage in the X-ray monitoring of the bone cement.^[22,23]^

## 5 Conclusions

Compared with the patients with intravertebral clefts OVCF, the pain relief was better in the patients without intravertebral clefts OVCF and less bone cement was injected into the surgery.

## Author contributions

**Funding acquisition:** Yingjie Zhou.

**Investigation:** Xubin Chai, Hanjie Zhuo.

**Methodology:** Hongxun Cui.

**Project administration:** Yingjie Zhou.

**Supervision:** Liang Kong, Wenxiao Zhu.

**Writing – original draft:** Dongfang Li.

**Writing – review & editing:** Dongfang Li.
